# Upper airway morphology in adults with positional obstructive sleep apnea

**DOI:** 10.1007/s11325-023-02879-0

**Published:** 2023-07-19

**Authors:** Xiaoxin Shi, Kate Sutherland, Frank Lobbezoo, Erwin Berkhout, Jan de Lange, Peter A. Cistulli, M. Ali Darendeliler, Oyku Dalci, Ghizlane Aarab

**Affiliations:** 1 Department of Orofacial Pain and Dysfunction, Academic Centre for Dentistry Amsterdam (ACTA), University of Amsterdam and Vrije Universiteit Amsterdam, Gustav Mahlerlaan 3004, 1081 LA Amsterdam, the Netherlands; 2 Department of Oral Radiology & Digital Dentistry, Academic Centre for Dentistry Amsterdam (ACTA), University of Amsterdam and Vrije Universiteit Amsterdam, Amsterdam, The Netherlands; 3 Department of Oral and Maxillofacial Surgery, Amsterdam University Medical Centers/Academic Centre for Dentistry Amsterdam (ACTA), University of Amsterdam, Amsterdam, The Netherlands; 4 Centre for Sleep Health and Research, Department of Respiratory and Sleep Medicine, Royal North Shore Hospital, Sydney, Australia; 5 Charles Perkins Centre and Northern Clinical School, Sydney Medical School, Faculty of Medicine and Health University of Sydney, Sydney, Australia; 6 Discipline of Orthodontics and Paediatric Dentistry, Sydney Dental School, University of Sydney, Sydney, Australia; 7 Department of Orthodontics, Sydney Dental Hospital, Sydney Local Health District, Sydney, Australia

**Keywords:** Positional obstructive sleep apnea, Cone beam computed tomography, Anatomical balance of the upper airway, Upper airway shape

## Abstract

**Purpose:**

To compare the anatomical balance and shape of the upper airway in the supine position between adults with positional obstructive sleep apnea (POSA) and adults with non-positional OSA (NPOSA).

**Methods:**

Adults diagnosed with OSA (apnea-hypopnea index (AHI) > 10 events/h) were assessed for eligibility. POSA was defined as the supine AHI more than twice the AHI in non-supine positions; otherwise, patients were classified as NPOSA. Cone beam computed tomography (CBCT) imaging was performed for every participant while awake in the supine position. The anatomical balance was calculated as the ratio of the tongue size to the maxillomandibular enclosure size. The upper airway shape was calculated as the ratio of the anteroposterior dimension to the lateral dimension at the location of the minimal cross-sectional area of the upper airway (CSAmin-shape).

**Results:**

Of 47 participants (28 males, median age [interquartile range] 56 [46 to 63] years, median AHI 27.8 [15.0 to 33.8]), 34 participants were classified as having POSA (72%). The POSA group tended to have a higher proportion of males and a lower AHI than the NPOSA group (*P* = 0.07 and 0.07, respectively). After controlling for both sex and AHI, the anatomical balance and CSAmin-shape were not significantly different between both groups (*P* = 0.18 and 0.73, respectively).

**Conclusion:**

Adults with POSA and adults with NPOSA have similar anatomical balance and shape of their upper airway in the supine position.

**Trial registration:**

This study was registered with the Australian New Zealand Clinical Trials Registry (ANZCTR Trial ACTRN12611000409976).

## Introduction

Obstructive sleep apnea (OSA) is characterized by recurrent complete (i.e., apnea) and partial (i.e., hypopnea) obstructions of the upper airway [1]. These respiratory events are most frequent and severe in the supine position [2]. It has been estimated that more than 50% of patients with OSA suffer from positional OSA (POSA) [3, 4]. Despite having well defined clinical features and specific treatment recommendations, the underlying mechanisms for POSA are not clear [5]. Evidence suggests that the differential pathogenesis between POSA and non-POSA (NPOSA) is partly attributable to anatomical factors, such as upper airway morphology [5, 6]. Some anatomical OSA treatments (e.g., upper airway surgery and oral appliance therapy) also show different treatment effects between patients with POSA and NPOSA [7, 8].

The upper airway is surrounded by soft tissue structures enclosed by maxillomandibular bony structures. Research suggests that both soft and hard craniofacial structures are related to upper airway shape and size, and these affect the severity of OSA [9]. The ratio of tongue size to maxillomandibular enclosure size – termed ‘anatomical balance’ – is an important risk factor in OSA, particularly in the supine position [10, 11]. Anatomical imbalance (i.e., an enlarged tongue size to maxillomandibular enclosure size ratio) indicates higher tissue pressure surrounding the upper airway and decreased upper airway size [12]. However, only a few studies have compared craniofacial structures between POSA and NPOSA groups. Two studies have suggested that patients with POSA may have a smaller lower facial height, more backward positioned mandible, and shorter soft palatal length than patients with NPOSA [13, 14]. However, Saigusa et al. [14] found no significant difference in the anatomical balance of the upper airway between the POSA and NPOSA groups in their study. Thus, studies on the differences in craniofacial structures between both groups are still limited.

Furthermore, studies by Pevernagie et al. [6] and Jiao et al. [13] have suggested that patients with NPOSA have a more circular cross-sectional area of the upper airway compared to patients with POSA, which may explain the differential pathogenesis between the groups. However, in both studies, participants had relatively severe OSA, and patients with NPOSA had more severe OSA. By contrast, a study by Joosten et al. [15] found no significant difference between POSA and NPOSA in upper airway shape, but the sample size was small (each group had only eight subjects). Hence, the difference in upper airway shape between POSA and NPOSA is not clear [5]. Accordingly, a large-scale study across the spectrum of OSA severity is necessary to provide more evidence on the potential differences in upper airway shape between POSA and NPOSA and their effect on treatment outcome.

In the supine position, the exacerbation of obstructive respiratory events may be related to the collapse of the anterior wall of the upper airway due to the effect of gravity. Accordingly, we hypothesize that: (1) adults with POSA have a greater anatomical imbalance (i.e., a higher ratio of tongue size to maxillomandibular enclosure size) compared to adults with NPOSA, and therefore a greater tendency of upper airway collapse in the anteroposterior direction in the supine position; and (2) adults with POSA have a more elliptically shaped cross-sectional area (i.e., a smaller ratio of the anteroposterior dimension to the lateral dimension) compared to adults with NPOSA, and therefore a greater tendency of upper airway collapse in the anteroposterior direction in the supine position. Accordingly, this study aimed to compare the anatomical balance and shape of the upper airway between adults with POSA and adults with NPOSA in the supine position.

## Material and methods

### Overview

This study is part of a prospective study [16], in which adults with OSA (apnea–hypopnea index (AHI) > 10 events/h) were recruited for building a prediction model for oral appliance treatment outcomes. This study received ethical approval (Sydney Local Health District, Protocol No. X11-0134 & HREC/11/RPAH/192), and informed consent was obtained from all participants. This study was registered with the Australian New Zealand Clinical Trials Registry (ANZCTR Trial ACTRN12611000409976).

### Participants

Patients were recruited prospectively from sleep clinics associated with a tertiary teaching hospital in Sydney (Royal North Shore Hospital). The inclusion criteria were adults (> 18 years of age) diagnosed with OSA (AHI > 10 events/h) using in-laboratory polysomnography (PSG) who were willing to try oral appliance therapy. Exclusion criteria were limited to contraindications to oral appliance therapy (e.g., severe periodontal disease and an insufficient number of teeth). There were no upper limits on AHI or body mass index (BMI) for recruitment. Ethnicity was recorded using a self-reporting questionnaire, in which participants selected their ethnicity based on their culture, religion, skin color, and language from the following four ethnic groups: Asian, Native Hawaiian and Pacific Islanders, Hispanic and Latino, and White. Patients were classified as positional OSA (POSA) if their AHI in supine position (AHI-supine) was more than twice their AHI in non-supine positions (AHI-non-supine) [17]. If this criterion was not fulfilled, patients were classified as non-positional OSA (NPOSA).

### CBCT acquisition

Every participant underwent a CBCT scan (NewTom 3G, QR systems, Italy) in the supine position while awake [16]. During the scanning process, patients were asked to bite gently in natural occlusion, lightly touch their teeth with their tongue, refrain from swallowing, and bring their lips into a relaxed position. The exposure settings were 110 kVp, 2-3 mA, and exposure times were 5–15 s. Voxel size was 0.36 mm or 0.42 mm, depending on the scan settings. A standardization of head position was performed after scanning. During this process, the palatal plane was adjusted to be parallel to the axial and sagittal planes and perpendicular to the coronal plane. CBCT datasets were saved as Digital Imaging and Communications in Medicine (DICOM) files.

### Anatomical balance

**Fig. 1:**
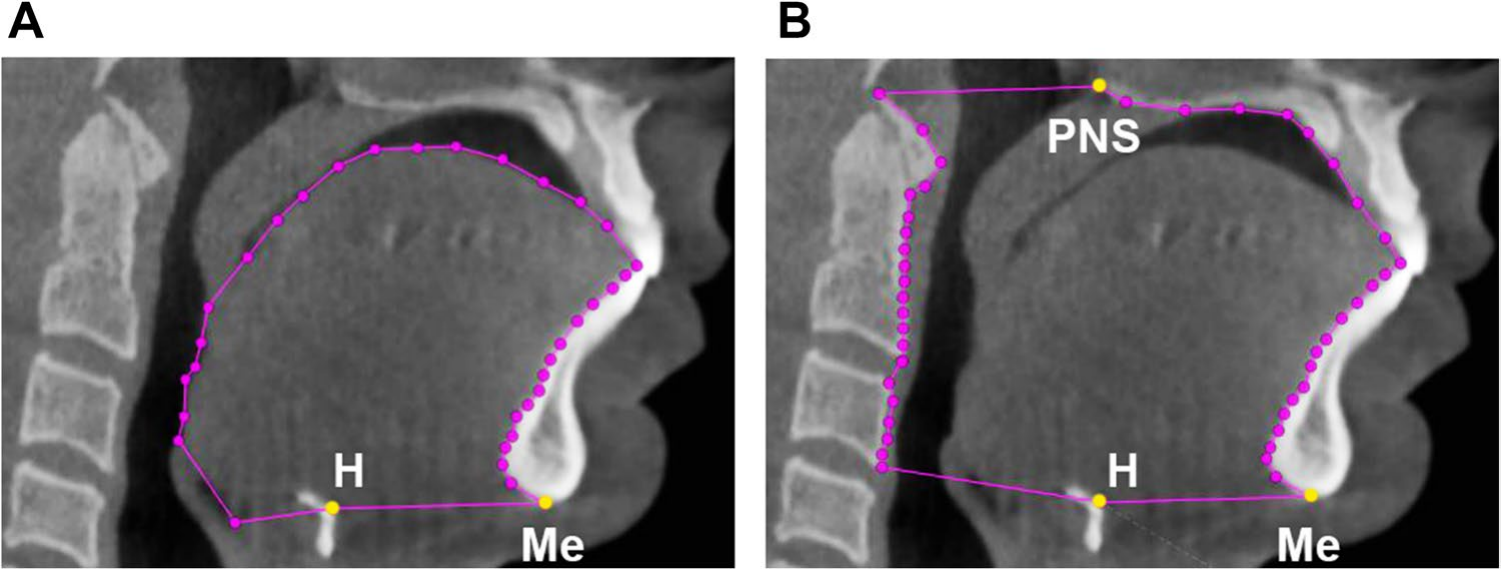
Measurements of tongue size and maxillomandibular enclosure size using cone beam computed tomography (CBCT) imaging in the mid-sagittal plane. (**A**) Tongue size: area enclosed by the point Hyoid (H), Menton (Me), the contour of the frontal teeth and tongue, and the base of the epiglottis. (**B**) Maxillomandibular enclosure size: area enclosed by the point Hyoid (H), Menton (Me), the contour of the front teeth, the hard palate, point posterior nasal spine (PNS), and the anterior boundary of the second and third cervical vertebra

**Fig. 2:**
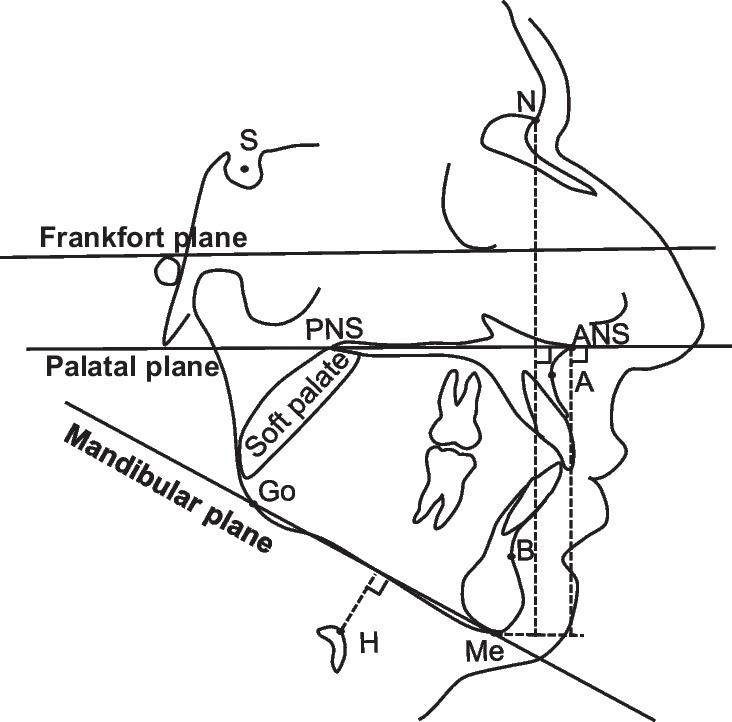
Illustration of the secondary outcome variables of the craniofacial structures. Soft palate length (distance from PNS to the tip of the soft palate), Hyoid to MP plane (distance between H and Mandibular plane), SNA (angle between points A and S at N), Maxillary length (distance between ANS and PNS), SNB (angle between points B and S at N), Mandibular length (distance between Me and Go), Mandibular plane angle (angle between Frankfort plane and Mandibular plane), Face height (distance between N and Me), Lower face height (distance between ANS and Me). Landmarks: N = nasion, S = sella, ANS = anterior nasal spine, PNS = posterior nasal spine, Go = gonion, Me = menton, H = hyoidale, A = subspinale, B = supramentale

3Diagnosys® software (v5.3.1, 3diemme, Cantu, Italy) was used to measure craniofacial structures, including the soft palate, tongue, hyoid, maxilla, mandible, and face height. The primary variable – the anatomical balance of the upper airway – was calculated as the ratio of the tongue size to maxillomandibular enclosure size in the mid-sagittal plane of the CBCT image. Tongue size was defined as the area enclosed by the point Hyoid (H), Menton (Me), the contour of the frontal teeth and the tongue, and the base of the epiglottis (Fig. 1A). Meanwhile, the maxillomandibular enclosure size was defined as the area enclosed by the point Hyoid (H), Menton (Me), the contour of the front teeth, the hard palate, the point posterior nasal spine (PNS), and the anterior boundary of the second and third cervical vertebra (Fig. 1B). The definitions for the secondary outcome variables, such as the soft palate, tongue, hyoid, maxilla, mandible, and face height, are illustrated in Fig. 2.

### Upper airway shape

**Fig. 3:**
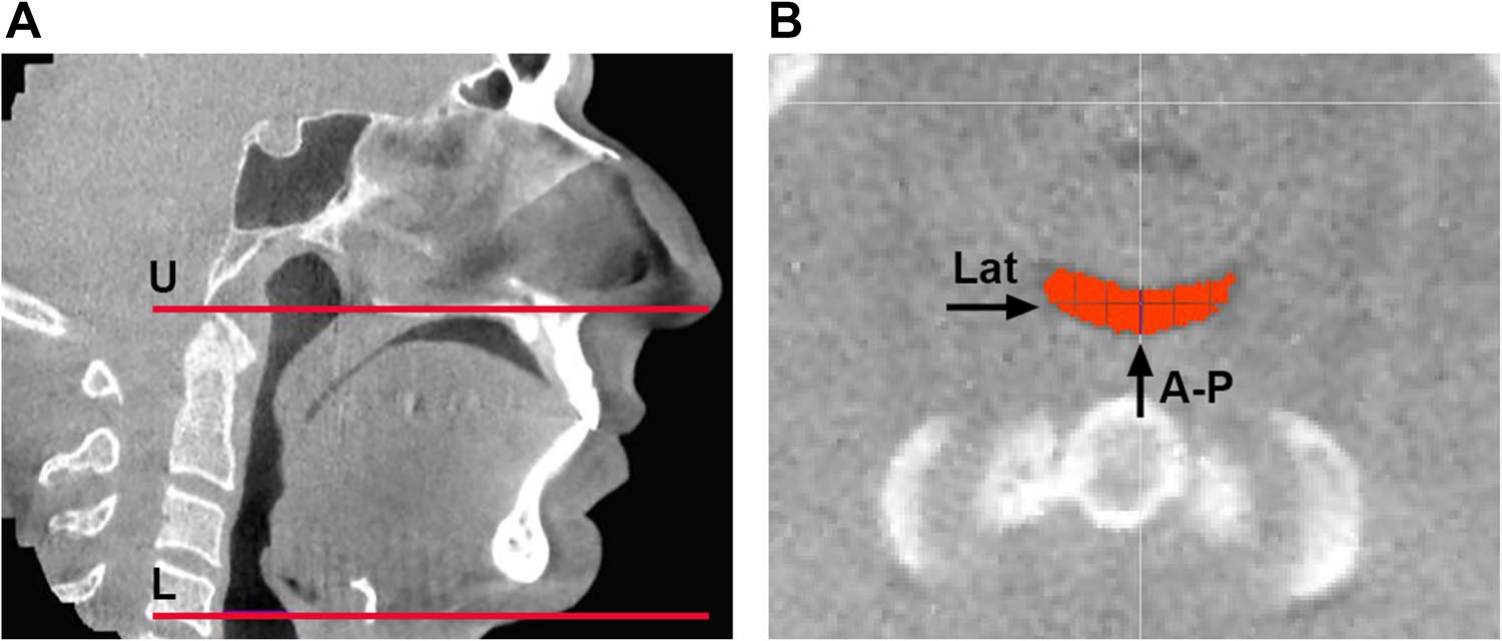
Upper airway boundaries and measurements of the upper airway shape, using cone beam computed tomography (CBCT) imaging. (**A**) The segmentation of the upper airway from the upper boundary (U) to the lower boundary (L) in the mid-sagittal plane. (**B**) The measurement of the anterior–posterior dimension (A-P) and lateral dimension (Lat) of the minimum cross-sectional area of the upper airway (CSAmin) in the axial plane

Upper airway shape and size were analyzed using CBCT images with Amira® software (v4.1, Visage Imaging Inc., Carlsbad, CA, USA). The superior boundary of the upper airway was the palatal plane; the inferior boundary was the horizontal plane (parallel to the palatal plane) across the base of the epiglottis (Fig. 3A). After applying the upper airway boundaries, the total upper airway volume (V) and the cross-sectional area (CSA) of each slice were calculated using the same software. The minimum CSA (CSAmin) was identified based on the CSA results. The anteroposterior dimension (A-P) and lateral dimension (Lat) of CSAmin were measured (Fig. 3B) on the specific slice where the CSAmin was located. The shape of CSAmin (CSAmin-shape) was calculated as the ratio of A-P to Lat; this was the primary outcome variable for representing the upper airway shape at the CSAmin location.

### Reliability of measurements

All upper airway variables were measured by an experienced examiner blinded to group membership. To determine the intra-observer reliability of the measurements, 15 CBCT scans were randomly selected and re-measured after three months of the original measurements.

The calculation of sample size for the reliability assessment was performed according to Walter et al. [18]. We assumed that our actual intra-observer reliability in the present study is at least 0.9, and a reliability of 0.6 or higher would be acceptable based on our previous study [19]. Therefore, the null hypothesis was defined as H0: ρ0 = 0.6, and the alternative hypothesis was defined as H1: ρ1 = 0.9, with a significance level of 0.05 and a power of 0.8. Based on this hypothesis, the proposed sample size was 15 patients [18].

### Statistical analysis

A Shapiro-Wilk test was used to test the normality of continuous data. An independent t test (for normally distributed variables), a Mann–Whitney U test (for non-normally distributed variables), and a Chi-squared test (or Fisher’s exact test) (for nominal variables) were used to compare the baseline demographic characteristics and PSG parameters between the POSA and NPOSA groups.

An intraclass correlation coefficient (ICC) was performed to determine the intra-observer reliability of the measurements of anatomical variables. The anatomical variables of upper airway morphology were compared between the POSA and NPOSA groups using analysis of covariance (ANCOVA). The baseline characteristics that were significantly different between both groups were treated as covariates. The significance level was set at 0.05, and statistical analyses were carried out using SPSS software (SPSS version 26, Chicago, IL, USA).

The effect sizes for primary outcome variables were calculated using the program G*power (version 3.1.9, Franz Faul, Universität Kiel, Germany).

## Results

### Participant’s recruitment

A total of 60 patients were assessed for eligibility. Of these, 13 patients with incomplete information were excluded from the analysis: *n* = 2 with incomplete baseline demographic data, *n* = 5 with incomplete PSG data, and *n* = 6 with CBCT images that were distorted or/and lacked landmarks for the primary outcome variables. After exclusions, 47 patients were included in the analysis: 34 patients with POSA, and 13 patients with NPOSA.

### Patient characteristics

The baseline demographic characteristics and respiratory and sleep parameters for POSA and NPOSA groups are provided in Table 1. There were no significant differences between the groups in age, sex, body mass index, neck circumference, waist circumference, and ethnicity (*P* = 0.07–0.88). For the respiratory variables, no significant differences were found in AHI and AHI-supine between both groups (*P* = 0.07 and 0.39, respectively); however, by definition, the AHI-non-supine was significantly higher in the NPOSA group compared to the POSA group (*Z* = −4.31, *P* < 0.01). For the sleep variables, there were no significant differences between the groups (*P* = 0.20–0.75). As the sex and AHI tended to have a significant difference between the groups (*P* = 0.07 and *P* = 0.07, respectively), these two variables were treated as covariates in the ANCOVA analyses.

**Table 1:** Baseline demographic characteristics, respiratory and sleep parameters of the POSA and NPOSA groups

	POSA (*n* = 34)	NPOSA (*n* = 13)	Test statistic	*P*
Demographic characteristics				
Age (years)	55.5 (46.0–63.3)	60.0 (46.0–63.0)	-0.16 (Z)	0.88
Sex (male vs female)	23 vs 11	5 vs 8	3.33 (χ^2^)	0.07
BMI (kg/m^2^)	29.2 ± 5.1	30.1 ± 6.2	-0.52 (t)	0.61
Neck circumference (cm)	40.5 ± 3.3	39.0 ± 3.2	1.34 (t)	0.19
Waist circumference (cm)	102.0 ± 12.6	100.2 ± 14.0	0.43 (t)	0.67
Ethnicity (race 1 vs 2 vs 3 vs 4)	7 vs 1 vs 3 vs 23	1 vs 0 vs 0 vs 12	2.53 (FET)	0.58
Respiration				
AHI (events/h)	21.8 (13.7–30.8)	32.2 (21.7–39.9)	-1.82 (Z)	0.07
AHI-supine (events/h)	44.0 (24.0–64.1)	34.8 (20.1–56.9)	-0.86 (Z)	0.39
AHI-non-supine (events/h)	6.0 (3.0–13.5)	30.7 (23.3–34.4)	-4.31 (Z)	< 0.01*
Sleep				
TST (min)	360.1 ± 45.0	330.8 ± 117.6	0.87 (t)	0.40
ST-supine (min)	165.0 ± 97.8	126.2 ± 75.5	1.29 (t)	0.20
ST-REM (%)^a^	15.8 ± 6.3	15.1 ± 7.3	0.32 (t)	0.75
ST-NREM (%)^b^	84.0 ± 5.2	84.9 ± 7.3	-0.43 (t)	0.67

Normally distributed data are shown as means ± standard deviations (SD); non-normally distributed data are presented as medians (interquartile range); t: independent t test; Z: Mann–Whitney U test; χ^2^: Chi-squared test; FET: fisher’s exact test

*POSA* positional OSA, *NPOSA* non-positional OSA, *BMI* body mass index, *Ethnicity* race 1 = Asian, race 2 = Native Hawaiian or Pacific Island, race 3 = Hispanic or Latino, race 4 = White, *AHI* apnea–hypopnea index, *AHI-supine* AHI in supine position, *AHI-non-supine* AHI in positions other than supine position, *TST* total sleep time, *ST-supine* sleep time in the supine position, *ST-REM* sleep time in rapid-eye-movement stage, *ST-NREM* sleep time in non-rapid-eye-movement stage

^a^2 patients in the POSA group with incomplete REM data were excluded from the analysis

^b^5 patients in the POSA group with incomplete NREM data were excluded from the analysis

^*^statistically significant

### Reliability of measurements

The intra-observer reliability was excellent for the primary variables (ICC = 0.91 for the anatomical balance, ICC = 0.97 for the CSAmin-shape) and secondary variables (ICC = 0.92–0.99) [20].

### Anatomical balance

**Fig. 4:**
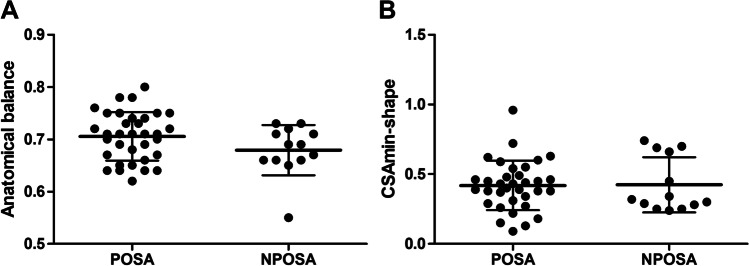
Individual values (dot plots, means with standard deviations) of the anatomical balance and CSAmin-shape of the POSA group (*n* = 34) and NPOSA group (*n* = 13). (**A**) Illustration of the anatomical balance of the upper airway. (**B**) Illustration of the CSAmin-shape

Table 2 provides the anatomical variables of the upper airway of the POSA and NPOSA groups. There was no significant difference between the groups for the primary outcome variable: the anatomical balance of the upper airway (*F* = 1.85, *P* = 0.18). The individual values of anatomical balance for both groups are shown in Fig. 4A. The effect size f of the anatomical balance comparison was 0.2 (partial *η*^2^ = 0.04, ANCOVA). This can be qualified as being between small and medium. For the secondary outcome variables, there were no significant differences between the groups either (all *P* > 0.05).

**Table 2:** The anatomical variables of the upper airway of the POSA and NPOSA groups

	POSA (*n* = 34)	NPOSA (*n* = 13)	F^a^ (ANCOVA)	*P*
Primary outcome variable				
Anatomical balance ratio	0.7 ± 0.0	0.7 ± 0.0	1.85	0.18
Secondary outcome variables				
Maxillomandibular enclosure area (mm^2^)	4666.7 ± 528.2	4403.4 ± 567.0	1.46	0.23
Tongue area (mm^2^)	3294.6 ± 413.5	2985.4 ± 377.4	3.71	0.06
Soft palate length (mm)	38.7 ± 4.2	40.1 ± 4.5	0.25	0.62
Hyoid to mandibular plane (mm)	18.8 ± 6.1	16.2 ± 4.6	0.90	0.35
SNA (°) ^b^	82.7 ± 3.2	81.5 ± 2.5	0.03	0.87
Maxillary length (mm)	55.1 ± 3.9	53.2 ± 3.2	1.30	0.26
SNB (°) ^b^	78.6 ± 4.0	77.6 ± 3.1	0.01	0.92
Mandibular length (mm)	70.5 ± 4.6	69.2 ± 4.0	0.09	0.76
Mandibular plane angle (°)	24.3 ± 6.1	26.2 ± 4.5	0.59	0.45
Face height (mm) ^b^	118.7 ± 6.0	116.5 ± 9.0	0.14	0.71
Lower face height (mm) ^c^	65.9 ± 4.7	65.3 ± 5.3	0.13	0.72

Data are shown as means ± standard deviations

*SNA* angle between sella, nasion, and subspinale (point A), *SNB* angle between sella, nasion, and supramentale (point B)

^a^the sex and AHI were controlled as covariates for the comparisons

^b^3 patients in the POSA group with incomplete CBCT image were excluded from the analysis

^c^1 patient in the POSA group with incomplete CBCT image was excluded from the analysis

### Upper airway shape

The upper airway dimensional variables of the POSA and NPOSA groups are shown in Table 3. For the primary outcome variable, CSAmin-shape, there was no significant difference between the groups (*F* = 0.12, *P* = 0.73). The individual values of the CSAmin-shape of both groups are presented in Fig. 4B. The effect size f of the CSAmin-shape comparison was 0.05 (partial η^2^ = 0.003, ANCOVA), which can be qualified as small. For the secondary outcome variables, there were no significant differences between the groups either (*P* = 0.23–0.98).

**Table 3:** The upper airway dimensions of the POSA and NPOSA groups

	POSA (*n* = 34)	NPOSA (*n* = 13)	F^a^ (ANCOVA)	*P*
Primary outcome variable				
CSAmin-shape	0.4 ± 0.2	0.4 ± 0.2	0.12	0.73
Secondary outcome variables				
A-P (mm)	4.9 ± 2.0	5.1 ± 1.4	0.18	0.68
Lat (mm)	12.8 ± 4.5	13.1 ± 3.5	1.51	0.23
CSAmin size (mm^2^)	60.2 ± 40.3	61.3 ± 20.1	0.48	0.49
L (mm)	66.6 ± 7.7	63.1 ± 8.1	0.63	0.43
V (cm^3^)	12.8 ± 11.9	12.0 ± 4.1	0.00	0.98

Data are shown as means ± standard deviations

*CSAmin* minimum cross-sectional area of the upper airway, *A-P* anterior–posterior dimension of the CSAmin, *Lat* lateral dimension of the CSAmin, *CSAmin-shape* the ratio of A-P to Lat, *L* upper airway length, *V* upper airway volume

^a^the sex and AHI were controlled as covariates for the comparisons

## Discussion

In the present study, we hypothesized that adults with POSA have a greater anatomical imbalance and a more elliptically shaped cross-sectional area of the upper airway in the supine position compared to adults with NPOSA. The results of this study showed no significant difference between both groups in these outcomes.

### Anatomical balance of the upper airway

No significant difference in the anatomical balance between the POSA and NPOSA groups was found. The observed effect size of the anatomical balance comparison was small to medium [21]. Therefore, the clinical relevance of the difference in this variable between both groups is controversial. Consistent with our results, Saigusa et al.’s study [14] calculated the anatomical balance using three-dimensional structures’ volume with magnetic resonance imaging (MRI) and found no significant difference between POSA and NPOSA either.

Regarding secondary variables, previous studies have indicated that patients with POSA show a larger ANB angle, smaller lower facial height, and shorter soft palate length compared to patients with NPOSA [13, 14]. However, we found no significant difference between both groups in those structures. Both previous studies’ samples were predominantly of Asian ethnicity, so the different ethnic balance in our study sample (74% white) may explain the different findings in these structures.

### Upper airway shape

The present study found no significant difference in CSAmin-shape between the POSA and NPOSA groups. Furthermore, the observed effect size of the CSAmin-shape comparison was small [21], indicating that the difference in CSAmin-shape between both groups is not clinically relevant. In contrast to our study results, a study by Pevernagie et al. [6] indicates that CSAmin-shape was more circular in the NPOSA group compared to the POSA group in the supine position. In their study, however, the NPOSA group had significantly higher AHI than the POSA group (83 vs. 31 events/h). Since it has been suggested that CSAmin-shape is related to AHI [22], the higher AHI in the NPOSA group could be a confounding factor in the comparison of CSAmin-shape, and it may explain the different results in comparison to our study. In line with our results, a study by Joosten et al. [15] suggested no significant difference in CSAmin-shape between both groups. Although their study had a small sample size (each group had eight subjects), our study confirmed their results with a larger sample. Therefore, it seems that upper airway shape is similar for POSA and NPOSA groups in the supine position, especially when their AHI severity is matched.

### Implications

As upper airway morphology was not significantly different between the POSA and NPOSA groups in the present study, it may be hypothesized that non-anatomical factors account for the differences in pathogenesis between both groups. Joosten et al. [23] have shown that, turning from a supine to a lateral position, the muscular ability to stiffen and dilate the airway is more effective in the POSA group compared to the NPOSA group. The less effective muscle activities in the NPOSA group may be caused by greater vulnerability to muscle fatigue (due to the increased number of type II fibers) [24], poor muscle effectiveness (caused by excessive fat or muscle hypertrophy) [25], and disturbed coordination of the neural drives between upper airway and respiratory muscles [26, 27]. Another study, undertaken by Joosten et al. [15], has suggested that lung volume decreases significantly in the POSA group when moving from the lateral to the supine position, while there is no significant change in lung volume with position changing in the NPOSA group. Decreased lung volume can increase upper airway collapsibility via caudal tracheal displacement [28, 29], which may be a trigger for upper airway collapse in the supine position in the POSA group [15]. However, these studies [15, 23] were performed either in only severe OSA samples or by comparing both groups with unmatched AHI. Therefore, whether POSA in comparison to NPOSA has its distinct pathophysiology characteristics still needs more research.

### Limitations

This study has several limitations. First, the patients were awake during the CBCT examination, which may not accurately reflect upper airway morphology during sleep. However, taking CBCT images during sleep is quite challenging, therefore, upper airway imaging in the awake state has been widely used to study the underlying pathogenesis of OSA [30]. As the CBCT images were taken in the supine position for both groups in the present study, we assume that the comparison results are valid. However, sleep-induced changes in upper airway morphology may be different in both groups. Therefore, future research performed in the sleep state is needed to verify the current results. Second, the CBCT measurements were collected as part of a previous treatment study [16] and, hence, only taken in the supine position. Images in non-supine positions would show whether the current non-significant findings are also applicable in other sleeping positions. Future research, including scans in both positions, is therefore recommended. Moreover, as this study recruited patients from a prospective study [16], we did not perform an a priori power analysis to calculate the sample size. However, the effect size of the primary outcome variables indicates that the differences in the anatomical balance and the CSAmin-shape between both groups are not clinically relevant. Third, the present study used Cartwright’s definition [17] to classify POSA, which is the most commonly used definition to date. However, compared to other definitions [31–34], Cartwright’s definition is the most lenient one and can result in a higher prevalence of POSA [32, 35]. Therefore, our study possibly overestimated POSA, which may explain the relatively high prevalence of POSA (72%) and the non-significant findings. However, we did also test our results with the stricter definition of the Amsterdam Positional OSA Classification (APOC) [32]. Although some patients who did not have sufficient sleep time in supine and/or non-supine positions were excluded from the classification by using the APOC definition (reducing the total sample size to *n* = 38), the prevalence of POSA was similar (APOC vs Cartwright: 71% vs 72%), and the comparison results of the primary outcome variables were similar to the present ones. Therefore, the criteria used in the current study did not lead to biased results. POSA tends to be associated with mild and moderate OSA as opposed to severe OSA [31], and the majority of this study population (62%) was diagnosed with mild to moderate OSA, which may explain the high prevalence of POSA in our population.

## Conclusions

Adults with POSA and adults with NPOSA have similar anatomical balance and shape of their upper airway in the supine position.

## Data Availability

The datasets generated and/or analyzed during the current study are available from the corresponding author on reasonable request.
